# Micro-/Meso-Structure Control of Multi-Hostmetal Alloys by Massive Nitrogen Supersaturation

**DOI:** 10.3390/ma17061294

**Published:** 2024-03-11

**Authors:** Tatsuhiko Aizawa

**Affiliations:** Surface Engineering Design Laboratory, Shibaura Institute of Technology, Tokyo 144-0045, Japan; taizawa@sic.shibaura-it.ac.jp; Tel.: +81-3-6424-8615

**Keywords:** low-temperature plasma nitriding, nitrogen supersaturation, two-phase nanostructure, microscopic solid-phase separation, nitrogen-rich clusters, nitrogen-poor clusters, chemical disturbance, masking technique, selective solid-phase separation

## Abstract

The low-temperature plasma nitriding was utilized to describe the microscopic solid-phase separation in the austenitic stainless-steel type AISI316, induced by the nitrogen supersaturation. This nitrogen supersaturated layer with the thickness of 60 μm had a two-phase nanostructure where the nitrogen-poor and nitrogen-rich clusters separated from each other. Due to this microscopic solid-phase separation, iron and nickel atoms decomposed themselves from chromium atoms and nitrogen solutes in this nitrogen supersaturated AISI316 layer. These microscopic cluster separation and chemical decomposition among the constituent elements in AISI316 were induced in the multi-dimensional scale by the plastic straining along the slip lines in the (111)-orientation from the surface to the depth of matrix. The nitrogen solute diffused through the cluster boundaries into the depth. With the aid of masking technique, this nitrogen supersaturation and nanostructuring was controlled to take place only in the unmasked AISI316 matrix. The nanostructures with two separated clusters were mesoscopically embedded into AISI316 matrix after the masking micro-textures. This microscopic and mesoscopic structure control was available in surface treatment of multi-host metals such as superalloys and high entropy alloys.

## 1. Introduction

**Figure 1:**
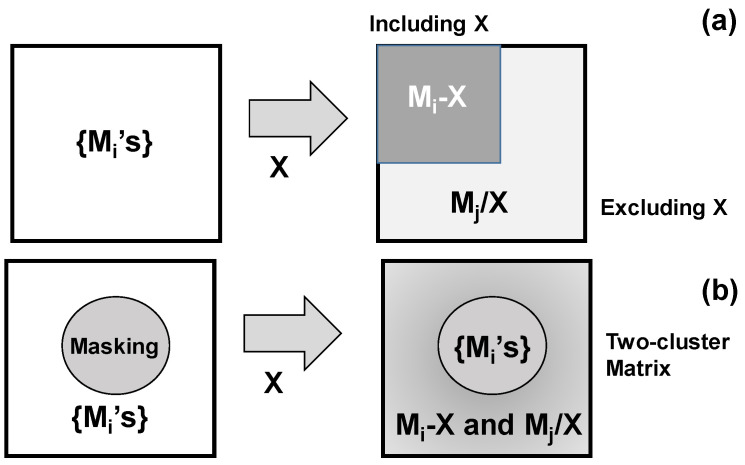
Solid-phase separation of multi-component alloy matrix by impinging the X-element solute. (**a**) Microscopic separation process to modify the original homogeneous matrix to two groups of clusters including or excluding the X-element, and (**b**) mesoscopic separation process to selectively modify the matrix by microscopic separation with aid of masking technique.

A multi-component alloy {M_1_, M_2_, …, M_n_} for constituent metallic components, Mi (1 < i < n), such as the high-entropy alloys [1], is sensitive to the thermal and stress transients. As theoretically stated in [2], it is decomposed into several binary and ternary alloy groups with their crystallographic structures during the post-treatments. As discussed in [3], except for decomposition in the high-entropy alloy nanoparticles, the high-entropy alloy solid is difficult to decompose in the atomic level to a group of separated Mi clusters in the solid state. This difficulty in the solid-phase separation of metallic alloys might come from insufficient driving force other than the thermal and stress transients. Consider that an element X is impinged into the targeting alloy system with different affinity of Mi to X. Then, as illustrated in Figure 1a, the original multi-component system {Mi} for 1 < I < n could separate itself to two groups of {Mi + X} clusters, including X and {Mj/X} clusters excluding X for i ≠ j. With aid of the masking technique, the masked region remains to have a multi-component system of {Mi’s} with no X-element contents. Meanwhile, the regions selectively including X-element with high content have two-cluster microstructure with Mi-X and Mj/X for i ≠ j, as depicted in Figure 1b.

The austenitic stainless-steel type AISI316 is employed as a model alloy with M_1_ = Fe, M_2_ = Ni and M_3_ = Cr. A nitrogen solute is employed as a X-element to drive this separation process, to demonstrate the possibility of microscopic and mesoscopic solid-phase separation processes in the multi-component metallic alloy by impinging the X-element solute with high content,

Nitrogen implantation was effective to impinge the nitrogen solute into the stainless-steel matrix [4]. Although the nitrogen supersaturated layer thickness is controlled by the acceleration energy of nitrogen ions, it is limited to be far less than 1 μm. Plasma nitriding provides a way to diffuse the nitrogen solutes into matrix [5]. This inner nitriding behavior is much dependent on the holding temperature. The conventional plasma nitriding processes at higher temperature than 700 K, such as ion-nitriding and radical-nitriding, so-called in market, were characterized by precipitation reaction of chromium and iron nitrides at the nitrided layer [6]. That nitride precipitation hindered the chemical interaction between Mi’s (1 ≦ i ≦ n) and nitrogen solutes in the matrix. In addition, the nitrogen content decayed exponentially from the surface to the depth and the nitrogen solute content was lower than 0.2 mass% [7]. Hence, the phase separation process has the risk of being significantly affected by the diffusing nitrogen solute content. At the low-temperature plasma nitriding, the nitrogen solute is impinged with its more content than the solubility limit into the AISI316 matrix without nitride precipitation [5]. As first stated in [8], this nitrogen supersaturation by the plasma nitriding, is characterized by the lattice expansion of γ-lattices in the austenitic stainless steels. This nitrogen supersaturation (NS) improves their corrosion toughness and hardness of the original austenitic stainless steels, as stated in [9,10]. As theoretically estimated in [11], their constituent elements are attracted to the nitrogen solutes. No chemical reactions took place between the carbon in the diamond chips and the iron in the NS-AISI316 matrix when machining the NS-AISI316 works by the PCD (Poly-Crystal Diamond)-chipped cutting tools [12,13]. Furthermore, as theoretically estimated and experimentally described in [5,14,15], this nitrided layer had uniform nitrogen content distribution from its surface to the nitriding front end. The average nitrogen content of this plateau reached 4 mass%.

In the present paper, the plasma nitrided AISI316 substrate at 673 K was prepared for microscopic and mesoscopic analyses on its NS-layer. The nitrogen solute distributes uniformly down to the depth of 60 μm. The lattice expansion of NS-supercells results in their elastic straining. Meanwhile, the other supercells neighboring them are plastically strained to compensate for the strain incompatibility at the nitriding front end. This plastic straining significantly modifies the polycrystalline structure to fine two-phase nanostructures, where the N-rich and N-poor clusters separate from each other. This microscopic solid separation induces the chemical decomposition among iron, nickel and chromium in the NS-AISI316 matrix. Iron and nickel, with less affinity to nitrogen, decompose from chromium with more affinity to nitrogen. With aid of the masking technique, the unmasked AISI316 specimen is nitrogen supersaturated to separate the original AISI316 matrix to embed the two-cluster structured nanostructure into the original matrix.

## 2. Materials and Methods

### 2.1. Plasma Nitriding System

**Figure 2:**
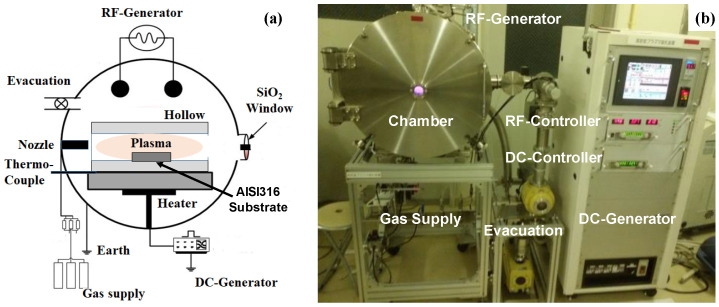
Low temperature plasma nitriding system for nitrogen supersaturation into AISI316 substrates. (**a**) Schematic view on the experimental setup, and (**b**) overview of experimental system.

The RF (Radio-Frequency)–DC (Direct Current) plasma nitriding system (YS-Electrical Industry, Co., Ltd.; Kofu, Japan) with the use of the hollow cathode was utilized to prepare the nitrogen supersaturated AISI316 (NS-AISI316) specimen for its microscopic and mesoscopic analyses, as schematically illustrated in Figure 2a. 

Owing to the hollow cathode device, the nitrogen ion and NH (nitrogen–hydrogen) radical densities were enhanced in the hollow as studied in [16]. After its plasma diagnosis, the ion density reached 4 × 10^18^ ions/m^3^ under the gas flow ratio of nitrogen gas by 160 mL/min to hydrogen gas flow by 20 mL/min. The RF–DC powers were automatically controlled with prompt response to the spatial impedance change in plasmas by adjusting the frequency of RF power as well as the DC-bias voltage.

In the following experiments in Figure 2b, the chamber was evacuated down to the base pressure of 0.1 Pa after setting the substrate into the hollow. Under the nitrogen atmosphere, the substrate temperature was increased up to 673 K by monitoring the thermocouple which was embedded into the base plate. After presputtering in the nitrogen atmosphere for 1.8 ks at 673 K, the AISI316 substrate was immersed in the nitrogen–hydrogen plasma at 70 Pa under the specified gas flow ratio at 673 K for 14.4 ks. After nitriding, the substrate was cooled down in the nitrogen atmosphere.

### 2.2. Selective Nitrogen Supersaturation with Aid of Masking Technique

**Figure 3:**
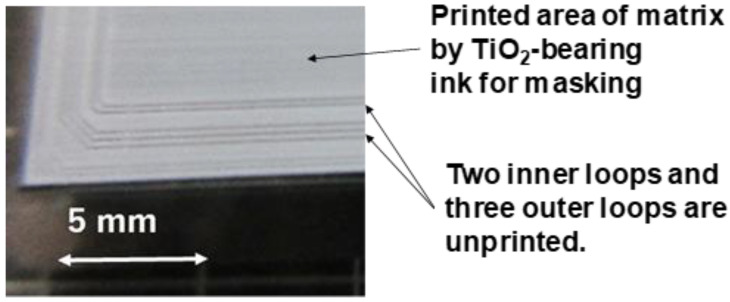
Screen printing on the AISI316 substrate surface for masking. Two inner loops and outer three loops were unprinted, while other surfaces were printed by the TiO_2_-bearing ink.

The screen printer was utilized to print the designed microtextures onto the AISI316 substrate as depicted in Figure 3. 

The screen film had a masked pattern with two inner loops and three outer loops. After printing through this screen, the whole substrate surface was all printed by the TiO_2_-bearing ink, except for these loops of mask on the screen film. Those printed patterns worked as a thermal resistant mask to make selective nitriding into those loops. On the other printed areas, the nitrogen ions and NH radicals were blocked to penetrate the matrix even at 673 K [17,18].

### 2.3. Microscopic and Mesoscopic Analyses on the NS-AISI3316 Layer

XRD (X-Ray Diffraction; Shimazu, Kyoto, Japan) was used to describe the γ-supercell expansion with two-phase formation on the nitrided AISI316 substrate surface at 673 K. SEM (Scanning Electron Miscopy; JOEL, Tokyo, Japan)–EDX (Electron Dispersive X-ray spectroscopy; JEOL, Tokyo, Japan) was also utilized to analyze the microstructure change by the nitrogen supersaturation, as well as the element mapping. EBSD (Electron Back Scattering Diffraction; JEOL, Tokyo, Japan) was used to describe the plastic straining, the phase distribution and the crystallographic orientation profile on the surface and cross-section of the NS-AISI316 substrate. A sample for EBSD analysis was made by cutting at its center after polishing its surface. Then, this cross-section was cleaned and polished by ion milling process with the use of argon ion beam. 

The KAM (Kernel Angle Misorientation) profile represents the equivalent plastic strain distribution from the surface to the depth. The phase mapping describes the phase transformation induced by the nitrogen supersaturation. IPF (Inverse Pole Figure) measures the grain size and orientation distribution.

In parallel to this mesoscopic analysis, STEM (Scanning Transmission Electron Microscopy; JEOL, Tokyo, Japan; JEM-ARM 200F) was used to make microscopic analysis at the atomic level. This Cs-corrected STEM attains high spatial resolution in 0.08 nm. In this STEM, the raster-scanned electron beam transmits through the specimen to provide three types of TEM images: LAADF (Low-Angle Annular Dark Field), HAADF (High-Angle Annular Dark Field) and ABF (Annular Bright Field) images. Three images, as well as electron diffraction X-ray analysis, were utilized to describe the solid-phase separation by nitrogen supersaturation and the chemical decomposition among Fe, Cr and nickel in the AISI316 matrix.

## 3. Results

The nitrogen supersaturation process was characterized by the γ-supercell expansion, the homogeneous nitrogen solute distribution with high content and the plastic straining. This nitrogen supersaturation induced the nano-structuring of the homogeneous γ matrix into a two-clustering nanostructure system with the chemical decomposition of the homogeneous Fe-Ni-Cr alloy system into Fe-Ni/N and Cr-N microscopic textures. The nitrogen solutes were embedded into the depth of the AISI316 substrate after microtextures for masking to form the NS-AISI316 microtextures, separate from AISI316 matrix.

### 3.1. Nitrogen Supersaturation into AISI316 Matrix

**Figure 4:**
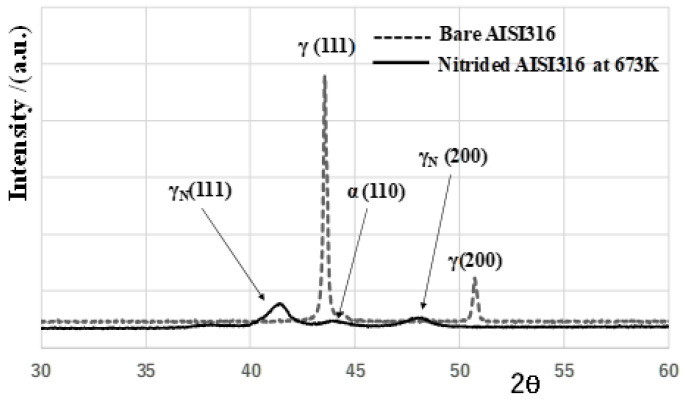
Comparison of XRD diagrams between the bare AISI316 matrix and the nitrided AISI316 at 673 K.

XRD was utilized to describe the phase change in AISI316 surface by the plasma nitriding at 673 K with reference to [19]. As shown in Figure 4, the original AISI316 before nitriding was characterized by γ-phase peaks at 2θ = 43.5° for γ (111) and at 2θ = 51° for γ (200), respectively.

This single-phase structure was modified by the plasma nitriding to be a two-phase structure with refined grain size. These γ (111) and γ (200) peaks shifted in the lower 2θ angle to γ_N_ (111) at 2θ = 41° and γ_N_ (200) at 2θ = 48°, respectively. That is, the original γ supercell in AISI316 matrix expanded by the nitrogen solute occupation into the octahedral vacancy sites of γ supercells, as theoretically predicted in [20]. In addition to these peak shifts, the α-phase peak was also detected as α (110) at 2θ = 44.5°. This implies that the original single-phase matrix separated into γ- and α-phase structures by the nitrogen supersaturation. To be noticed, these γ_N_ and α peaks were significantly broadened; after [21], the original supercells in AISI316 matrix were refined into nano-sized grains both for γ_N_-phase and α-phase structures.

**Figure 5:**
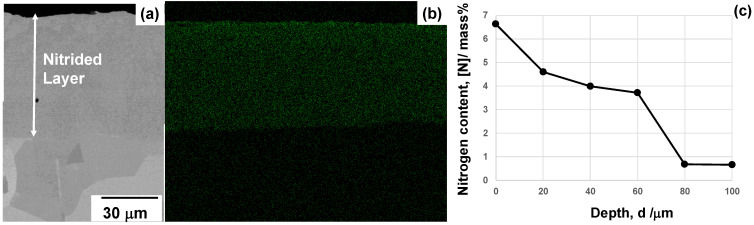
SEM–EDX analysis on the cross-section of nitrided AISI316 substrate at 673 K. (**a**) SEM image on the cross-section including the nitrided layer, (**b**) nitrogen mapping from the surface to the depth across the nitriding front end and (**c**) nitrogen solute content depth profile.

SEM–EDX analysis was employed to describe the cross-sectional microstructure and nitrogen mapping of the NS-AISI316 layer from its surface to the depth across the nitriding front end. As depicted in Figure 5a, b, the nitrogen solute distributed homogeneously in the NS-AISI316 layer with the thickness of 60 μm. 

After the pointwise analysis by EDX, the average nitrogen solute content reached 4 mass% in Figure 5c. This plateau with high nitrogen content remained into the depth until the vicinity of the nitriding front end.

The γ-supercell expansion induced its lattice strain in the NS-AISI316 zones from the surface. The un-nitrided zones neighboring to these NS zones were subjected to elastic straining by this lattice strain. Then, those un-nitrided zones were plastically strained to compensate for the strain incompatibility across the NS and un-nitrided zone boundaries. EBSD was first utilized to describe this plastic straining behavior induced by the nitrogen supersaturation.

**Figure 6:**
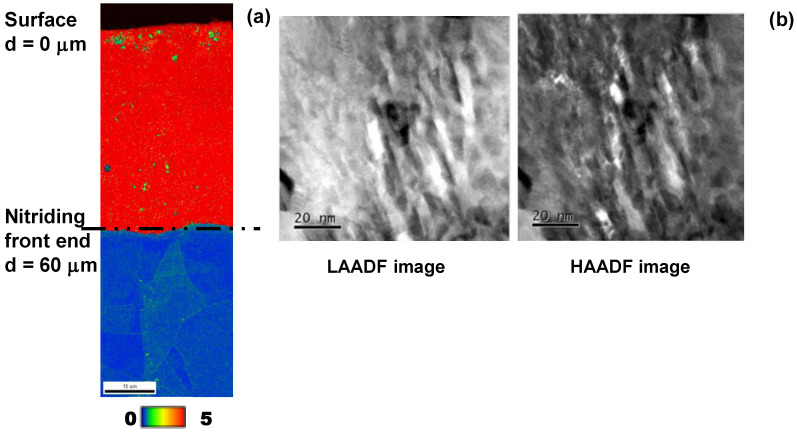
The slip-line system induced into AISI316 matrix by the plastic straining during the nitrogen supersaturation from the surface to the depth. (**a**) KAM distribution into the depth of matrix, and (**b**) LAADF and HAADF images by the STEM observation on the middle depth in the NS-AISI316 layer.

The KAM profile is depicted in Figure 6, which represents the equivalent plastic strain distribution after [22].

The whole NS-AISI316 layer had the highest misorientation angle; this proves that it was significantly plastically strained. After the theory on the plasticity [22], this high plastic straining accompanies the shear localization to form the slip-line field. Both LAADF and HAADF images are shown in Figure 6b. 

Nano-sized slip-lines were aligned with the skew angle of 45° in the vertical direction from the surface to the depth. This proves that these dense slip-line field in the atomic level is detected as a high misorientation angle layer in the mesoscopic analysis by EBSD in Figure 6a. This regular and dense slip-line field was continuously formed from the surface to the depth in the NS layer. With nitrogen solute transportation through the dense slip-line filed network, the NS zones advanced with elastic straining by their lattice expansion and accompanied plastic straining in the neighboring supercells to the nitriding front end. Hence, a regular network of slip-lines must have been left on the lateral surface of NS-AISI316 layer. Let us describe this regularity in the slip-line field formation at the vicinity of NS-AISI316 surface. 

**Figure 7:**
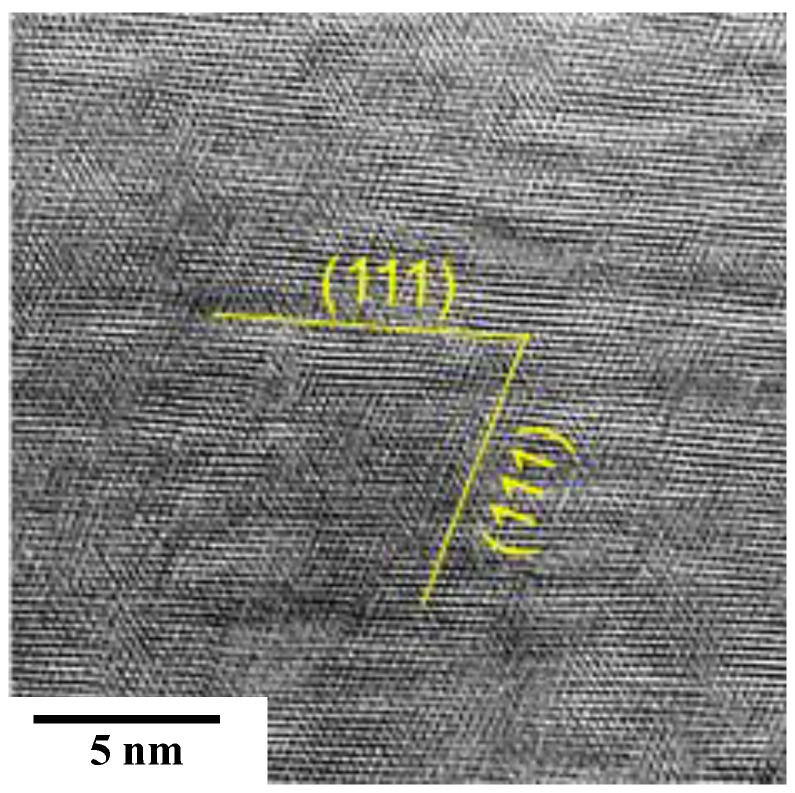
The STEM observation and analysis on the plastically strained nanostructure at the vicinity of the NS-AISI316 layer surface.

Figure 7 depicts the ABF image at the vicinity of the NS-AISI316 surface by STEM analysis.

The original polycrystalline microstructure was completely modified into a nano-granular structure. Every cluster zone with the size of 5 nm was regularly aligned to have common crystallographic orientation to (111). Hence, the NS-AISI316 layer at the vicinity of its surface became a single crystal with this specific crystallographic orientation.

Figure 6 and Figure 7 reveal that the slip-line field was continuously formed from the surface layer to the depth in the NS-AISI316 layer. As shown in Figure 6a, KAM distributed even below the nitriding front end. Then, the original microstructure below the NS-AISI316 layer with high nitrogen solute content must have been affected by the plastic straining via the nitrogen supersaturation.

**Figure 8:**
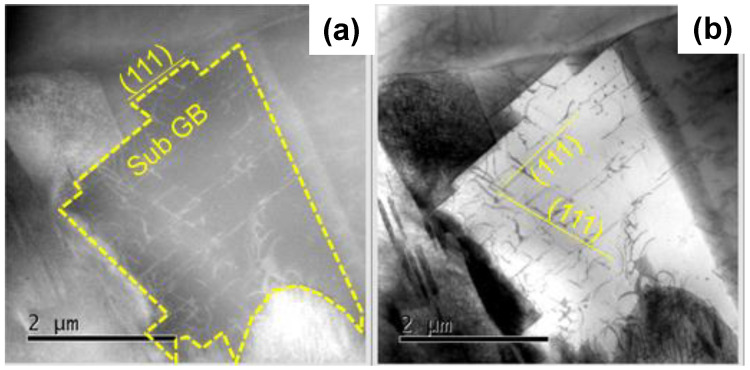
The STEM analysis on the slip-line system induced into the depth of AISI316 matrix at d = 60 μm below the nitriding front end by the plastic straining. (**a**) HAADF image, and (**b**) ABF image.

Figure 8a depicts a typical modified crystal with the sub-grain-boundary in the crystallographic orientation of (111). 

This proves that the original microstructure was plastically deformed to align its sub-grain-boundaries along the slip-line field. In addition, as shown in Figure 8b, the inner grains were also aligned along the orientation of (111). This fine network of slip-line field in Figure 8 corresponds to the high KAM distribution in each grain below the nitriding front end in Figure 6a. This slip-line field network worked even below the nitriding front end as a nitrogen solute diffusion path to continue the microstructure modification through the nitrogen supersaturation.

**Figure 9:**
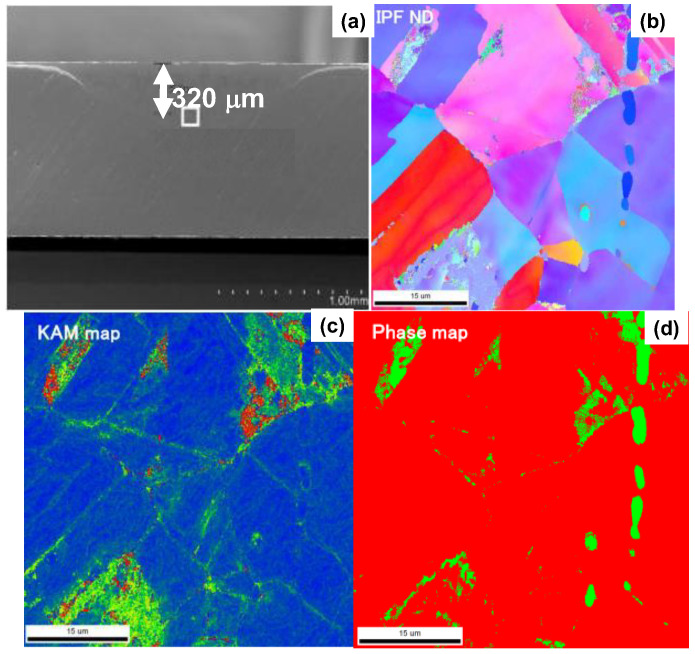
The EBSD analysis on the slip-line system induced into the far depth of 320 μm from the surface of AISI316 matrix by the plastic straining. (**a**) SEM image, (**b**) IPF distribution, (**c**) KAM map and (**d**) phase map.

A sample for EBSD analysis was prepared to describe the microstructure change at far depth by 320 μm from the surface, as depicted in Figure 9a.

The IPF distribution, KAM and phase maps are also shown in Figure 9b, Figure 9c and Figure 9d, respectively. The grain boundaries were highly strained to have co-orientation to (111). Several crystals were also strained to build up small sub-grains in them. These crystallographic modifications revealed that the plastic straining worked as a first step to make nanostructuring of original AISI316 matrix through the nitrogen supersaturation.

### 3.2. Microscopic Solid-Phase Separation in the NS-AISI316 Matrix

**Figure 10:**
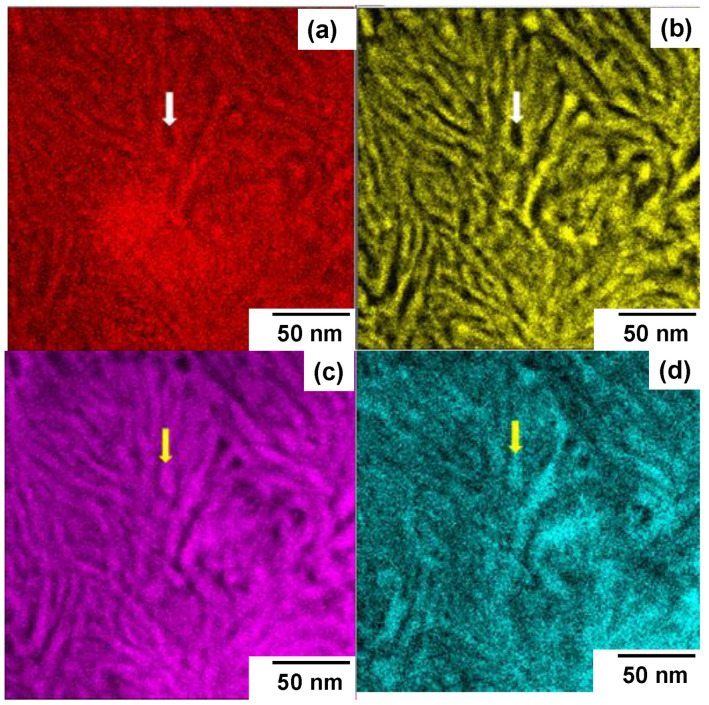
Chemical decomposition among the iron, nickel and chromium in the nitrogen supersaturated layer. (**a**) Microscopic iron map, (**b**) microscopic nickel map, (**c**) microscopic chromium map and (**d**) nitrogen solute map.

The nitrogen solutes diffused through the fine network of slip-line field and super-saturated the AISI316 sub-grains to modify the microstructure. The nanoscopic structure of constituent elements in the local supercells is described using the microscopic element mapping. The iron, nickel, chromium and nitrogen maps are compared among four elements in Figure 10.

The homogeneous element distribution before nitriding was completely modified to form the patterned textures. The iron and nickel atoms distributed in the same pattern where both are present at the absence of nitrogen solutes, while the chromium atoms coexisted in the same pattern with the nitrogen solute. This chemical decomposition was induced by the difference in affinity among Fe, Ni and Cr against the nitrogen solute atom. 

**Figure 11:**
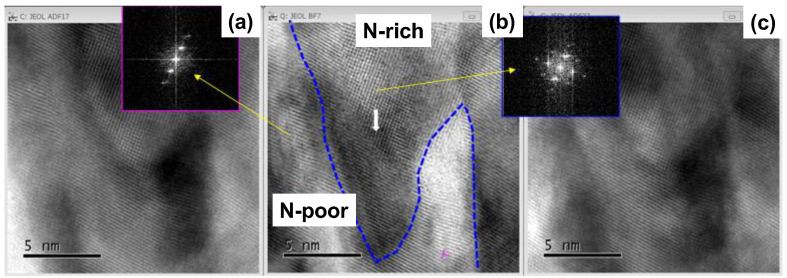
Microscopic solid-state separation from the homogeneous AISI316 matrix to the two clustered nanostructures by nitrogen supersaturation. (**a**) HAADF image, (**b**) ABF image and (**c**) LAADF image. Two N-rich and N-poor clusters were neighboring to each other along the slip-line field, induced by the plastic straining in the NS-AISi316 matrix.

Figure 11 depicts the HAADF, ABF and LAADF images on the patterned texture within the width of 5 nm in the NS-AISI316 matrix.

The N-rich cluster, which mainly consisted of chromium and nitrogen solute, is neighboring the N-poor cluster, which mainly consisted of iron and nickel. To be noticed in the electron diffraction pattern, each cluster had different crystallographic structure. That is, the N-rich clustering nanostructure had a bcc structure with higher nitrogen solute content in local, while the N-poor clustering nanostructure had a fcc structure with lower nitrogen solute content. Remember that the NS-AISI316 layer had two-phase nanostructures as analyzed by EBSD [5,15]. This separation into two-clustering nanostructures with different crystallographic structure, shown in Figure 11, is just corresponding to the formation of two-phase structuring as observed in EBSD analysis.

### 3.3. Selective Separation of AISI316 Matrix for Microtexturing by Nitrogen Supersaturation

The nitrogen supersaturation homogeneously started from the surface and advanced into the depth of AISI316 matrix with nitrogen solute transportation via the slip-line field network. When masking the surface by the designed pattern, this nitrogen supersaturation only took place from the unmasked surface areas into the depth.

**Figure 12:**
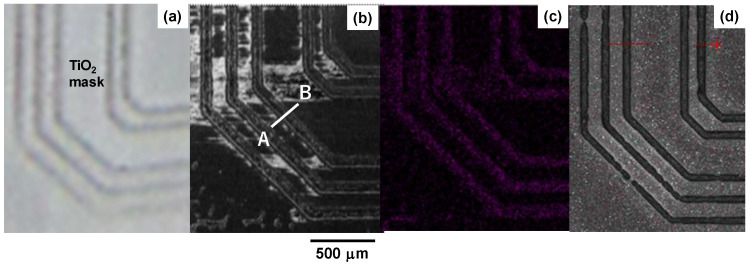
Selective separation for microtexturing the AISI316 matrix via the nitrogen supersaturation. (**a**) Printed mask pattern onto the AISI316 specimen surface, (**b**) selective nitrogen supersaturation into the unmasked regions, (**c**) nitrogen solute mapping and (**d**) mechanical removal of masked regions (red line denotes for height measurement).

The TiO_2_-bearing solvent was utilized as a thermo-resistant printing ink at the nitriding temperature to mask the AISI316 substrate surface as depicted in Figure 12a.

The white areas on the surface are a masked zone by TiO_2_-bearing ink, while the triplet and doublet black loops with each width of 50 μm were only unmasked to leave the bare AISI316 surfaces. The SEM image on the masked AISI316 substrate after nitriding at 673 K for 14.4 ks under the same plasma nitriding conditions is shown in Figure 12b. The nitrogen mapping in Figure 12c proves that unmasked loop-areas were only nitrided with high nitrogen solute content. That is, the NS-AISI316 loop domains with the thickness of 60 μm were embedded into the original AISI316 matrix. This selective separation of the NS microtextures from the bare AISI316 substrate was controllable by the microtexture design for masking.

**Figure 13:**
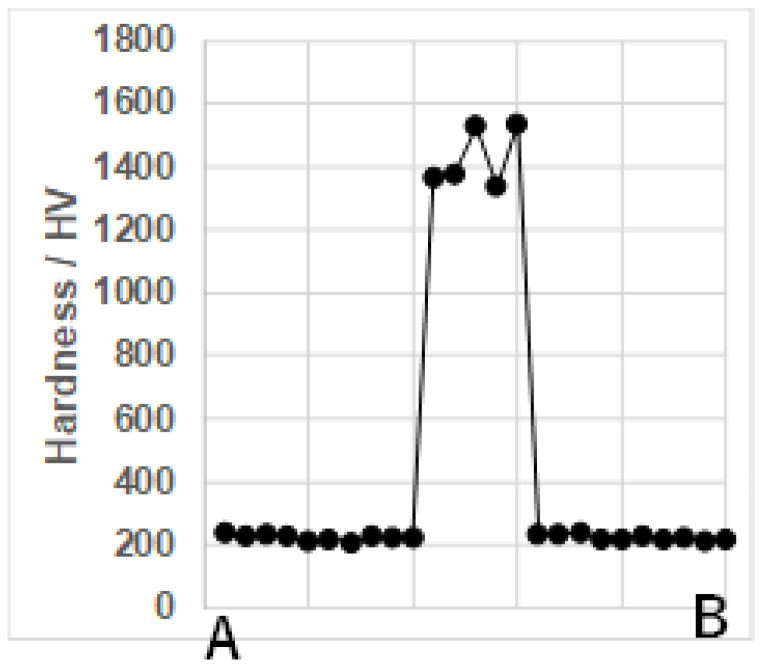
Hardness profile from A to B in Figure 12b across the nitrogen supersaturated zone.

The hardness profile was measured from A to B in Figure 12b and depicted in Figure 13 to demonstrate that the nitrogen mapping in Figure 12c is corresponding to the hardness change.

The masked areas were un-nitrided to have a bare matrix hardness of 200 HV, while the un-masked areas were nitrogen-supersaturated to have a high hardness of 1400 HV in average. Then, the masked areas were expected to be selectively removed using the silica shooting media with the hardness of 700 HV. Figure 12d demonstrates that the triplet and doublet loop heads with the height of 60 μm were formed by this mechanical blasting process, as expected.

## 4. Discussion

The low-temperature plasma nitriding provides a way to make massive nitrogen supersaturation (NS) into multi-host metal alloys. As depicted in Figure 5, the NS layer with the average nitrogen solute content of 4 mass% or 20 at% was formed to have the thickness of 60 μm by the plasma nitriding at 673 K for 14.4 ks. This nitrogen solute distribution with a high content plateau cannot be explained either by the normal diffusion process through the AISI316 matrix [7] or by the theoretical modification of diffusion mechanism [23]. As analyzed in Figure 6, Figure 7, Figure 8 and Figure 9, the formation of slip-line field by plastic straining during the massive nitrogen supersaturation process played a key to yield a network of nitrogen solute transportation from the surface to the far depth of the AISI316 matrix. As analyzed in Figure 8, the original grain boundaries were modified to sub-grain boundaries in (111), and the slip-line field formation in the inside of the grains demonstrated that a fine network of nitrogen solute diffusion paths was prepared even below the nitriding front end. Considering that the plastic straining was continuously induced just below the nitriding front end in Figure 8 and Figure 9, the NS layer above the nitriding front end included a massive nitrogen solute content to drive the further nitrogen supersaturation process in depth.

Let us consider the nanostructuring behavior in this massively nitrogen supersaturated (MNS) AISI316 matrix. As depicted in Figure 7 and Figure 11, the most of MNS-AISI316 matrix at the vicinity of surface is modified to be a single crystal, homogeneously including the high content nitrogen solutes by intense plastic straining at the vicinity of its surface. In almost all the NS layers except for the vicinity of surface, the nitrogen solute attracts the constituent element in the supercell with the orientation of (111). This attractive force to chromium atoms becomes the highest; it lowers against the iron atoms and becomes the lowest against the nickel atoms after the theoretical study in [11]. Then, the chemical decomposition is caused by the difference of attractive forces to the nitrogen solutes among three constituent elements. Due to this chemical decomposition, the supercell separated to the N-rich cluster, with a higher attraction of chromium to nitrogen solutes, as well as the N-poor cluster with a lower attraction of iron and nickel to nitrogen solutes in Figure 11. These two nanostructures are alternatively formed across the cluster boundaries to form the local deviation patterns among Cr, Fe and Ni contents in the MNS-AISI316 matrix.

Without masking to shield the nitrogen impinging from the plasma sheath to the AISI316 substrate surface, its whole layer was homogeneously nitrogen supersaturated with the chemical decomposition and two-cluster nanostructuring. Using the masking technique, this nitrogen supersaturation process was controlled to take place selectively only onto the unmasked zones. In case of masking in Figure 3, the AISI316 substrate surface was masked to leave two inner loops and three outer loops unmasked. Then, these loops were selectively nitrogen supersaturated to have sufficient hardness not to be removed mechanically by the sand-blasting as shown in Figure 12d and Figure 13. This revealed that the two-cluster nanostructured and chemically decomposed AISI316-base alloy was embedded into the bare AISI316 matrix after the printed microtextures for masking.

In the present study, AISI316 matrix was employed as a model material to describe the role of intense nitrogen impinging to make nanostructure separation and chemical decomposition. In almost all the multi-component alloys, each constituent metallic atom has its intrinsic chemical compatibility to the light interstitial element such as hydrogen, boron, carbon, nitrogen and oxygen. As reviewed in [14,24], this chemical interaction between the host transition metals and the light interstitial atoms changed the catalytic performance. The magnetic properties were significantly improved using the interstitial elements into multi-host metal alloys [25]. This massive interstitial solid solution effect on the mechanical properties and performance was also reported in [26]. TiNbZr with oxygen interstitial atoms by 12 at% had high compressive strength [27]. Those previous studies revealed that the mechanical and functional properties of high-entropy alloys (HEA) were significantly improved by massive interstitial supersaturation. The massive nitrogen supersaturation via the plasma nitriding at 673 K provided an efficient procedure to significantly improve the mechanical and functional properties of high entropy alloys.

In particular, two self-organization processes are attractive to control the mechanical and functional properties of MNS high entropy alloys: two-clustering nanostructuring and chemical decomposition. The homogeneous distribution of host metals is self-organized to deviate from each other to form the separated nanotextures. Magnetic and non-magnetic metals decompose themselves with the distance of 5 nm, as predicted by Figure 10 and Figure 11, resulting in the formation of a new family of metal materials [28]. Regular alignment of two nanocrystalline clusters is also attractive to improve the mechanical properties. In the case of MNS-AISI316, this solid-phase separation occurs under the high nitrogen solute content condition. As partially discussed in [5,15], higher nitrogen content than 3 mass% is needed to drive the interaction between the host metals of Cr, Fe and Ni and the nitrogen interstitial atom. Higher hardness than 1400 HV in [5] and high machinability of NS-AISI316 by the PCD chip in [12,13] were attributed to this high nitrogen interstitial content in host metals.

In a similar manner to embed the MNS-AISI316 textures into the bare AISI316 matrix, MNS-HEA is also built into the original HPA matrix after the designed texture. The regularly aligned NS-HEA is effective to control the functional properties of HEA. Higher nitrogen solute content than 15 at% in the supersaturated HEA is attractive to investigate the massive interstitial saturation effect on the improvement of various properties of HEA and related composites.

AISI316 die has been used as a mold die for hot stamping the oxide glass lenses [29]. MNS-AISI316 is expected to advance the mold-fie life due to its high hardness and strength by grain-size refinement. AISI316L and AISI316LN have been also utilized as a structural part without the magnetism and with the well-defined surface condition for future nuclear fusion reactor under the ITER-project [30]. MNS-AISI316L and MNS-AISI316LN are expected to improve the strength and hardness with sufficient anti-radiation capacity in addition to their very low magnetism and fine surface condition.

## 5. Conclusions

The solid-phase separation process of multi-component alloy via the nitrogen supersaturation (NS) is characterized by three steps: (1) plastic straining of alloy matrix to form the fine slip-line network for nanostructuring; (2) chemical decomposition of original homogeneous alloy matrix into two nano-clustering textures, including and excluding the nitrogen solutes; and (3) nitrogen diffusion through the cluster boundaries and along the fine slip-lines. Through this separation, the original AISI316 matrix was modified to decompose into the iron-nickel pattern with less nitrogen content and the chromium pattern with more nitrogen content. Each super cell of this decomposed NS-AISI316 consisted of nitrogen-poor and nitrogen-rich nanocrystalline structures. With the aid of the masking technique, this NS-AISI316 microtextures were embedded into the original AISI316 matrix after the masking pattern. The hardness was stepwise enhanced only on this NS-AISI316 textures across the boundaries between the NS-AISI316 microtextures and the AISI316 matrix, in correspondence to the enrichment of nitrogen content.

Nitrogen supersaturation provides a procedure to control the surface microstructure of super alloys and high-entropy alloys. Self-organization via the chemical decomposition at the atomic level and the nano-clustering by nitrogen enrichment improves the mechanical properties of NS-alloys. The selective nitrogen supersaturation with aid of masking technique is also effective to embed the NS alloy microtextures into the original alloy matrix.

## Data Availability

Not applicable.
